# Oligodendrogenesis in Evolution, Development and Adulthood

**DOI:** 10.1002/glia.70033

**Published:** 2025-05-15

**Authors:** Hao Hu, Tianhao Gao, Jingwei Zhao, Huiliang Li

**Affiliations:** ^1^ Wolfson Institute for Biomedical Research, Division of Medicine, Faculty of Medical Sciences University College London London UK; ^2^ Systemic Medicine Centre, School of Basic Medicine Zhejiang University School of Medicine Hangzhou China

**Keywords:** adaptive myelination, development, evolution, maladaptive myelination, myelin, oligodendrocyte, oligodendrogenesis

## Abstract

Oligodendrogenesis and myelin formation are important processes in the central nervous system (CNS) of jawed vertebrates, underpinning the highly efficient neural computation within the compact CNS architecture. Myelin, the dense lipid sheath wrapped around axons, enables rapid signal transmission and modulation of neural circuits. Oligodendrocytes are generated from oligodendrocyte precursor cells (OPCs), which are widely distributed in the adult CNS and continue to produce new oligodendrocytes throughout life. Adult oligodendrogenesis is integral to adaptive myelination, which fine‐tunes neural circuits in response to neuronal activity, contributing to neuroplasticity, learning, and memory. Emerging evidence also highlights the role of oligodendrogenesis in specialized brain regions, linking oligodendrocytes to metabolic and homeostatic functions. In the aging and diseased brain, dysregulated oligodendrogenesis exacerbates myelin loss and may contribute to pathogenesis. In addition, maladaptive myelination driven by aberrant neuronal activity could sustain a dysfunction in conditions such as epilepsy. This review summarizes the current understanding of oligodendrogenesis, with insights into its evolution, regulation, and impact on aging and disease.

## Main Points

- Oligodendrocytes and myelin took shape in jawed vertebrates
- During development, OPCs are originated from NSCs and then undergo differentiation into mature oligodendrocytes that supply myelin.
- OPCs persist in the adult CNS and continue to produce oligodendrocytes and myelin.
- Adaptive myelination, which fine‐tunes neural circuits in response to neuronal activity, contributes to neuroplasticity, learning and memory.
- Maladaptive myelination, driven by aberrant neuronal activity, could sustain a dysfunction in conditions such as epilepsy.

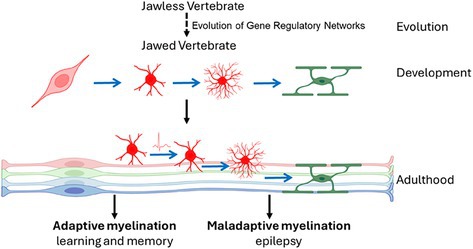

## 1 Introduction

The vertebrate nervous system is a marvel of complexity, where electrical activity within interconnected networks of neurons governs daily activities, behavior, and cognition (Swanson and Lichtman 2016). Information passes through these networks along axons, which transmit electrical impulses known as action potentials (APs). The speed of AP transmission is largely determined by whether axons are ensheathed, fully or partially, by myelin—the spirally wrapped, lipid‐rich membrane surrounding axons. Myelin allows rapid saltatory conduction, increasing AP transmission speed by more than 100‐fold (Zalc and Colman 2000). The emergence of myelin in jawed vertebrates marked a defining moment in vertebrate evolution, enabling higher‐order brain functions such as learning and memory (Li and Richardson 2008, 2016).

**FIGURE 1:**
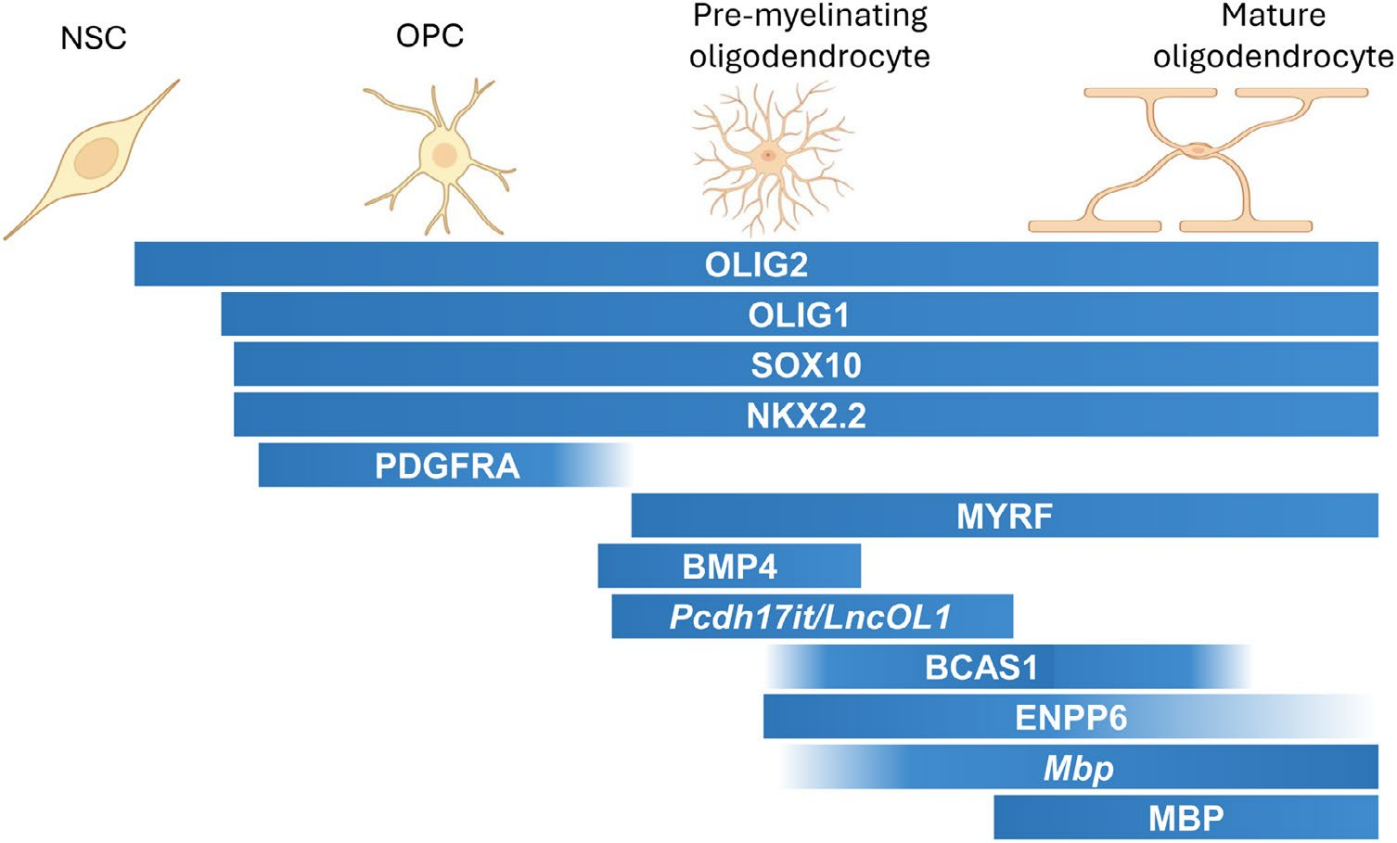
Oligodendrocyte lineage progression. OPCs originate from NSCs during neural development. These OPCs differentiate into pre‐myelinating oligodendrocytes, which subsequently mature into fully functional oligodendrocytes. Each stage is characterized by the expression of specific molecular markers.

Myelin is supplied by Schwann cells in the peripheral nervous system (PNS) and oligodendrocytes in the central nervous system (CNS). Like most neural cell types, oligodendrocytes are derived from neural stem cells (NSCs, or neural epithelial cells) in the developing neural tube (Richardson et al. 2006). During development, oligodendrocyte lineage cells progress through a series of stages, beginning with the specification of oligodendrocyte precursor cells (OPCs) from embryonic NSCs around mid‐gestation, followed by OPC proliferation, OPC migration to their resting sites in response to local environmental cues, OPC differentiation into immature (pre‐myelinating) oligodendrocytes, and further maturation into myelinating oligodendrocytes, culminating in the formation of myelin sheaths (Figure 1). In mammals, the majority of mature oligodendrocytes are generated in the early postnatal period (Psachoulia et al. 2009; Young et al. 2013).

Unlike other neural precursor cells, OPCs are widely distributed in the adult CNS, comprising approximately 5% of all neural cells in mice, and continue to proliferate and differentiate into mature oligodendrocytes throughout adulthood, albeit at a declining rate with age (Kasuga et al. 2019). While the precise roles of newly formed adult oligodendrocytes are still under investigation, they appear to be involved in processes beyond simple cell replacement. Oligodendrocytes are remarkably stable and long‐lived in the cerebral cortex and major white matter tracts (Fornasiero et al. 2018; Hughes et al. 2018; Kasuga et al. 2019; Simons et al. 2024; Tripathi et al. 2017; Yeung et al. 2014), minimizing the need for new oligodendrocytes to replace those damaged during normal cell turnover. Emerging evidence suggests that adult‐born oligodendrocytes play an integral part in activity‐dependent adaptive myelination, which involves myelinating previously unmyelinated axons, extending existing myelin sheaths, and remodeling the myelin structure. Adaptive myelination is thought to play a significant role in neuroplasticity, learning, and memory (Baraban et al. 2016; Bechler et al. 2018; Simons et al. 2024).

However, adult oligodendrogenesis may have drawbacks under certain conditions. For example, it has been linked to the shortening of myelin internodal length with age, potentially leading to excessive myelin production, disruption to myelin homeostasis, and reduction in axonal signal conduction speed. These changes could correlate with cognitive decline in aging (Bowley et al. 2010; Lasiene et al. 2009; Peters 2009; Young et al. 2013). In addition, impaired activity‐dependent myelination has been implicated in pathological conditions such as Alzheimer's disease (AD) (Chen et al. 2021; Wang et al. 2020), while maladaptive myelination, triggered by aberrant neuronal activity, may reinforce such activity, contributing to neurological disorders such as epilepsy and movement disorders (Atkinson‐Clement et al. 2020; Knowles, Batra, et al. 2022; Knowles, Xu, et al. 2022; Simons et al. 2024).

In this review, we outline recent progress in understanding oligodendrogenesis, focusing on its evolution, regulation, and impact on aging and disease.

## 2 Evolution of Oligodendrogenesis

During the Cambrian explosion, likely driven by two rounds of whole‐genome duplication (Dehal and Boore 2005; Yu et al. 2024), newly evolved vertebrates adapted to their environments through natural selection and by enhancing the velocity and efficiency of AP propagation (Nishiyama et al. 2021). This adaptation facilitated faster responses regarding catching prey and evading predators, crucial to survival. The emergence of myelin in evolution represented a pivotal innovation, allowing massively increased AP propagation speed without necessitating a proportional increase in axon diameter (Hartline and Colman 2007). This step in evolution is of crucial importance to optimizing neural signaling efficiency with a compact nervous system and accommodating the spatial constraints of vertebrate anatomy. Although in some invertebrates and jawless vertebrates, “ensheathing glial cells” were present to supply a “myelin‐like” membrane structure around axons, the first species to be equipped with compact, modern myelin sheaths were jawed vertebrates (Nave and Werner 2021; Verkhratsky et al. 2022; Weil et al. 2018; Zalc 2016; Zalc et al. 2008).

### 2.1 Schwann Cell Genesis Versus Oligodendrogenesis: Which Came First Evolutionarily?

The emergence of myelin in evolution is thought to have coincided with the appearance of a hinged jaw in placoderms (extinct), the oldest jawed vertebrates (Zalc and Colman 2000). As both the jaw structure and Schwann cells originate from neural crest cells, it has been speculated that PNS myelin might have evolved first. However, there is no fossil evidence to support that placoderms were hemi‐myelinated in the PNS. Moreover, the concept of hemi‐myelination seems improbable since some axons such as motor neuron axons traverse both the CNS and PNS, which would create a troubling discrepancy between the CNS and PNS in conduction speed along the neuronal pathways (Zalc 2006). An alternative hypothesis posits that the demand of vertebrate evolution for rapid AP transmission drove pre‐existing ensheathing glial cells to develop the ability to enwrap axons, simultaneously forming myelin in both the CNS and PNS (Zalc 2006, 2016).

In zebrafish, motor exit point (MEP) glia, which are CNS‐derived (from spinal cord OPCs) and express CNS identity markers Olig2 and Nkx2.2, have been found to migrate out of the CNS to myelinate the peripheral face of the MEP (Fontenas and Kucenas 2018, 2021; Smith et al. 2014). This observation lends credence to the “motor glia” hypothesis of myelin evolution, suggesting that oligodendrogenesis might have occurred first. According to this hypothesis, during early vertebrate evolution, some motor neurons—sharing the same precursors as oligodendrocytes in the gnathostome spinal cord—underwent reprogramming of gene expression to activate myelin‐related genes, resulting in the formation of “motor glia” that evolved into today's oligodendrocytes (Li and Richardson 2008, 2016; Richardson et al. 1997). Once the myelinating program began to evolve, the genetic and molecular mechanisms underpinning myelin production in oligodendrocytes could have been co‐opted by other glial cells given the appropriate environmental and developmental cues (Li and Richardson 2016; Zalc 2016). Notably, MEP glia share developmental properties with Schwann cells, employing mechanisms associated with neural crest cells in development (Fontenas and Kucenas 2018, 2021). In addition, some oligodendrocytes, such as those in the optic nerve, possess exceptionally long axon‐like processes (Young et al. 2013), adding weight to the idea of a possible evolutionary link between oligodendrocytes and motor neurons.

### 2.2 Evolution of Gene Regulatory Networks Underpinning Oligodendrogenesis

The evolution of gene regulatory networks is marked by their expansion and adaptation to incorporate novel genes and functions (Voordeckers et al. 2015). In particular, the duplication of transcription factor genes, coupled with mutations in their DNA‐binding regions or interaction domains, has driven the divergence of existing networks and the emergence of new regulatory pathways (Voordeckers et al. 2015). The gene regulatory networks specifying oligodendrocyte characteristics have recently been reviewed, with several evolutionary models being proposed to explain the origin of oligodendrogenesis (Hines 2021). Key transcription factors that are important to oligodendrocyte specification include basic helix–loop–helix (bHLH) proteins Olig1/Olig2 (Lu et al. 2002; Zhou and Anderson 2002) and SRY‐related HMG‐box E (SoxE) proteins Sox8/Sox9/Sox10 (with functional redundancy) (Finzsch et al. 2008; Stolt et al. 2005). Of these, Olig1/Olig2 are considered the upstream determinants (Hines 2021; Li and Richardson 2008; Stolt et al. 2006). Genomic analysis reveals that Olig2 orthologs are present in jawless vertebrates, such as hagfish (e.g., *Eptatretus burgeri* , Ensembl genes ENSEBUG00000012811 and ENSEBUG00000005107; *Myxine glutinosa* , NCBI gene 137428417) and lampreys ( *Petromyzon marinus* , NCBI gene 116955626), but their expression and function in these agnathans have yet to be explored (Lara‐Ramirez et al. 2019). Interestingly, members of the SoxE protein family have been detected in the developing neural tube of lampreys (Yuan et al. 2018), along with another important transcription factor for oligodendrocyte development, Nkx2.2 (Fu et al. 2002; Qi et al. 2001), implying that some elements of the oligodendrogenesis programme might have been present in early vertebrates, though how these networks were co‐opted and expanded to give rise to modern oligodendrocytes remains unresolved. Further study on gene regulation in lampreys and hagfish, as well as in chondrichthyes (the oldest extant jawed vertebrates), is essential to elucidating the origin and early evolution of oligodendrogenesis.

### 2.3 Pdgfra‐Negative OPCs: Functional Implications

Pdgfra and NG2 are widely recognized as molecular markers of OPCs, with their expression being directly regulated by Olig2 and SoxE transcription factors (Finzsch et al. 2008; Gotoh et al. 2018; Nishiyama et al. 1996; Pringle et al. 1992). In tetrapods, Pdgfra expression is critical to OPC proliferation and survival (Fu et al. 2002; Liao et al. 2022; Pringle et al. 1992). But in zebrafish, despite the presence of the *Pdgfra* gene, their OPCs notably lack *Pdgfra* expression (Mora 2006; Nakamura et al. 2012; Nishiyama et al. 2021; Park et al. 2002). Based on data from single‐cell RNA sequencing, although zebrafish OPCs do not express *Pdgfra*, a subset of them expresses *Cspg4* that encodes NG2 (Marisca et al. 2020). In rodents, evidence points to the existence of an OPC subpopulation that is positive for PLP1/DM‐20 but lacks PDGFRA expression (Spassky et al. 2000; Timsit et al. 1995). These cells are non‐proliferative and possibly represent pre‐myelinating or immature oligodendrocytes rather than true OPCs (Fruttiger et al. 1999; Richardson et al. 2000). Recent studies using advanced genetic tools have confirmed the existence of PDGFRA‐negative OPCs in the hindbrain of mice (Zheng et al. 2018). It is plausible to hypothesize that these PDGFRA‐negative OPCs may have an evolutionary origin tracing back to bony fish. Oligodendrocytes derived from such OPCs may primarily myelinate the neural circuits responsible for basic physiological functions, while those produced by the proliferative PDGFRA‐expressing OPCs likely target the neural circuits associated with higher‐order brain activities. Further research is needed to test this hypothesis and understand the distinct roles of these OPC subpopulations in brain function and evolution.

### 2.4 Evolution of Myelin: Potential Contribution of Ancient Retroviruses

The expression of myelin component genes in early gnathostomes in evolution required the recruitment and adaptation of novel regulatory networks (de Bellard 2016; Gould et al. 2008; Inouye and Kirschner 2016; Li and Richardson 2008, 2016; Nave and Werner 2021; Nawaz et al. 2013; Schweigreiter et al. 2006). Most myelin component proteins are believed to have been repurposed from pre‐existing genes, while some, for example, *myelin basic protein* (*Mbp*), appear to have emerged *de novo*, as evidenced by the lack of *Mbp* homologs in genomes of jawless vertebrates (though not yet fully sequenced) (Hines 2021; Nave and Werner 2021).

Recently, *RetroMyelin*, a retrotransposon RNA of retroviral origin, has been identified in jawed vertebrates as a key regulator of Mbp expression via a Sox10‐mediated mechanism (Ghosh et al. 2024). *RetroMyelin* plays an essential role in myelin production across mammals, amphibians, and fish. Phylogenetic analysis of *RetroMyelin* sequences from 22 jawed vertebrate species has revealed greater sequence similarity within species than between species, suggesting that *RetroMyelin* might have been independently acquired multiple times through the process of convergent evolution (Ghosh et al. 2024).

### 2.5 Crucial Events in the Evolution of Oligodendrogenesis

**FIGURE 2:**
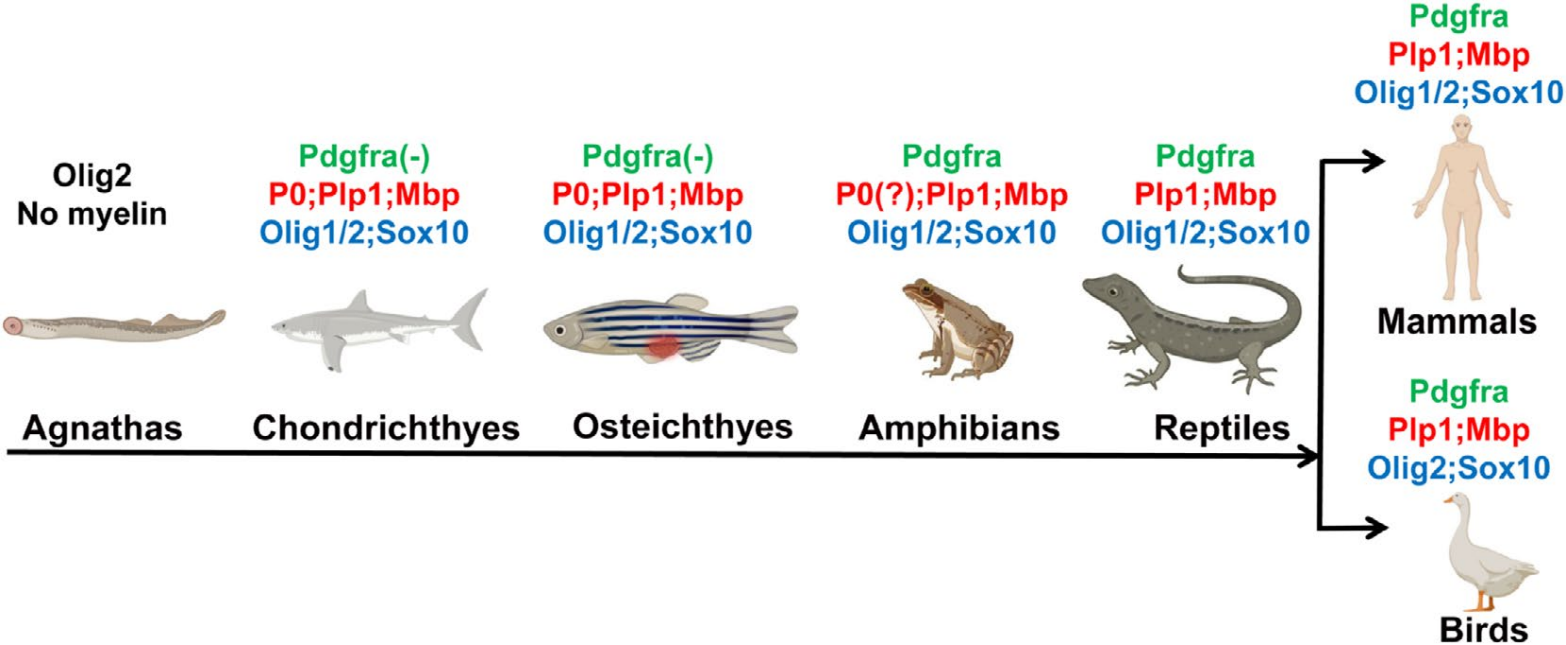
Evolution of Oligodendrogenesis. In early vertebrate evolution, agnathans had not evolved myelin, with only Olig2 expressed in the CNS. In jawed fish, including chondrichthyans and osteichthyans, oligodendrocyte‐associated transcription factors such as Olig1 emerged, and Pdgfra‐negative OPCs of simple developmental origin could differentiate into oligodendrocytes marked by the expression of myelin proteins such as Mbp, Plp1, and P0. In amphibians, OPCs began to express Pdgfra; although P0 expression was restricted to the adult PNS, it was present in the CNS of tadpoles. Reptiles evolved gene expression profiles in oligodendrocyte lineage cells similar to those of mammals. Birds missed Olig1, which is critical to remyelination. Genes expressed in OPCs, oligodendrocytes, and oligodendrocyte lineage cells are written in green, red, and blue, respectively.

According to the “motor glia” hypothesis, oligodendrocytes originated from motor glia, a specialized glial population sharing a developmental origin with motor neurons. This transition would have required the evolution of a new gene regulatory network orchestrated by key transcription factors such as Olig2 and SoxE proteins, enabling the reprogramming of motor glia into OPCs. Jawed fish started to be armed with Pdgfra‐negative OPCs of relatively simple developmental origin, which could differentiate into oligodendrocytes marked by the expression of myelin proteins such as Mbp, Plp1, and P0 (Park et al. 2002; Preston et al. 2019; Siems et al. 2021). In Xenopus, OPCs began to express Pdgfra (Mora 2006), signifying a developmental divergence. Interestingly, P0 expression was found to be restricted to the PNS in adult bullfrogs but present in the CNS of tadpoles (Takei and Uyemura 1993), implying an evolutionary transition in myelin protein localization. Reptiles evolved gene expression profiles in oligodendrocyte lineage cells comparable to those of mammals, while birds missed the *Olig1* gene (Li and Richardson 2016), which is critical to remyelination (Arnett et al. 2004). These crucial events in oligodendrogenesis evolution highlight how oligodendrocyte regulatory networks evolved and adapted to meet the diverse needs of vertebrate species (Figure 2).

## 3 Developmental Oligodendrogenesis

In early embryogenesis, NSCs from specific domains of the ventricular zone generate OPCs under the influence of local molecular cues (Lu et al. 2000; Richardson et al. 2006; Tekki‐Kessaris et al. 2001; Zhou et al. 2000). OPCs are stimulated by cellular motility factors to exit the ventricular zone, migrating along the vascular scaffold, guided by long‐range chemoattractants and chemorepellents, and settling into their final resting sites under the influence of local cells and microenvironmental cues, which altogether build a “biofield” for OPCs (Duncan and Emery 2023; Su et al. 2023; Tsai et al. 2016; Xia and Fancy 2021). Upon reaching their destinations, OPCs proliferate and differentiate into myelinating mature oligodendrocytes. This developmental process is tightly regulated by transcription factor networks, epigenetic factors, microRNAs, long non‐coding RNAs, and extracellular signals including growth factors and the extracellular matrix (ECM). Comprehensive reviews from numerous research groups have analyzed a variety of studies on the regulators governing oligodendrocyte development and the consequences of their dysregulation in developmental neurological disorders (Cristobal and Lee 2022; Dermitzakis et al. 2022; Elbaz and Popko 2019; Emery and Wood 2024; Motavaf and Piao 2021; Negah et al. 2025; Ngo and Kothary 2022; Parras et al. 2020; Rawani et al. 2024; Selcen et al. 2023; Sock and Wegner 2019; Xia and Fancy 2021; Yellajoshyula et al. 2022; Zhang et al. 2023).

### 3.1 Revisiting OPC Developmental Origin

In the developing mouse spinal cord, approximately 85% of OPCs originate from the motor neuron precursor (pMN) domain of the ventricular zone, while dorsal domains contribute around 15% of OPCs (Cai et al. 2005; Fogarty et al. 2005; Vallstedt et al. 2005). In the developing forebrain, OPC origins are more complex. Fate‐mapping studies with Cre‐Loxp based transgenic tools indicate that OPCs in the embryonic telencephalon are generated in three distinct waves—first wave (E12.5): OPCs are generated from NKX2.1‐positive progenitors in the medial ganglionic eminence (MGE) and anterior entopeduncular area (AEP) of the ventral forebrain; second wave (E15.5): OPCs are derived from GSH2‐positive progenitors in the lateral ganglionic eminence (LGE) and/or caudal ganglionic eminence (CGE); third wave (neonatal period): OPCs are originated from progenitors in the cortex (Chapman et al. 2013; Kessaris et al. 2006). Initially, it was believed that first‐wave OPCs were largely eliminated during early postnatal development (Kessaris et al. 2006; Richardson et al. 2006), but later studies revealed that a subpopulation of first‐wave OPCs survived and integrated into functional networks, forming specific clusters with interneurons (Orduz et al. 2019).

**FIGURE 3:**
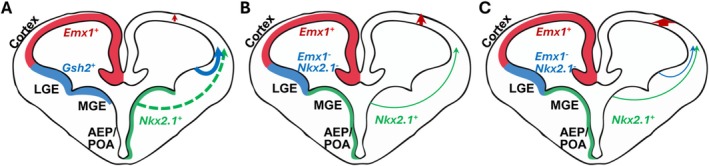
Models of OPC developmental origin in mouse cortex. (A) The first wave of OPCs arises from the medial ganglionic eminence (MGE), anterior entopeduncular area (AEP), and preoptic area (POA) in the ventral forebrain, followed by a second wave generated from the lateral ganglionic eminence (LGE), and a third wave originating postnatally within the developmental cortex. In this model, while MGE‐ and AEP/POA‐derived OPCs are eventually eliminated during development, LGE‐derived OPCs persist as the predominant source of OPCs and oligodendrocytes in the adult cortex (Kessaris et al. 2006). (B) Cortical OPCs and oligodendrocytes predominantly originate from MGE/AEP/POA as well as local cortical progenitors during development. This model reveals only minimal contribution from LGE to the adult cortical OPC/oligodendrocyte population (Li et al. 2024). (C) Progenitors within the developmental cortex are the primary source of cortical OPCs and oligodendrocytes in this model. While MGE and AEP/POA progenitors make a small but stable contribution to cortical OPCs, LGE‐derived cells primarily contribute to the piriform cortex (Cai et al. 2024).

However, recent studies have challenged the traditional three‐wave theory of telencephalic OPC generation (Figure 3). Using in utero electroporation to selectively label LGE‐derived OPCs, it was found that these OPCs predominantly produced striatal oligodendrocytes rather than contributing to cortical OPCs, and OPC migration from the LGE to the developing cortex was not detected (Li et al. 2024). Further evidence obtained from a triple transgenic mouse line (*Nkx2.1‐Cre; Emx1‐Cre; H2B‐GFP*) indicated no significant contribution of LGE/CGE to cortical OPCs (Li et al. 2024). Moreover, intersectional and subtractional fate‐mapping employing both Flp and Cre recombinases demonstrated that although LGE/CGE‐derived OPCs dominated regions such as the piriform cortex and anterior commissure, they contributed minimally to neocortex and corpus callosum OPCs (Cai et al. 2024). In addition, MGE‐derived OPCs, previously thought to be eliminated, were shown to make a small but sustained contribution to the cortical OPC pool (Cai et al. 2024). The discrepancies compared to previous findings could potentially be attributed to unfaithful or undetected recombinase (e.g., Cre) expression as well as recombinase efficiency in oligodendrocyte lineage cells within the transgenic lines used. Such variability in recombinase activity could lead to incomplete or inaccurate labeling of specific OPC populations, thus influencing the interpretation of their developmental origins. Furthermore, it should be noted that commonly used oligodendrocyte lineage specific markers such as PDGFRA, OLIG2, SOX10, OPALIN, and ASPA may not be uniformly expressed across all defined stages of oligodendrocyte lineage cells as previously acknowledged in this field (Kitada and Rowitch 2006; Marques et al. 2016; Zheng et al. 2018). Due to these limitations, current contradictory findings may only be able to be resolved through technological advancement, for example new in vivo imaging techniques for observing the developing cortex.

Nevertheless, studies suggest that oligodendrocytes of different developmental origins partially compensate for each other while retaining distinct functional roles. For instance, in the mouse spinal cord and corpus callosum, both ventrally and dorsally derived OPCs and mature oligodendrocytes exhibited similar electrophysiological properties but differentiating preferences for myelinating different axon populations (Tripathi et al. 2011). In response to demyelinating lesions, dorsally derived OPCs showed enhanced proliferation and differentiation compared to their ventrally derived counterparts in young adult mice (Crawford et al. 2016), and this regenerative capacity seemed to decline with age as those dorsally derived OPCs displayed reduced differentiation potential in aged mice (Crawford et al. 2016). Employing diphtheria toxin fragment A (DTA) for genetic ablation of dorsally derived oligodendrocytes resulted in defective myelination accompanied by locomotor and cognitive deficits (Foerster et al. 2024), despite compensatory myelination by ventrally derived oligodendrocytes in the adult mouse cortex. In addition, disrupting differentiation of dorsally derived OPCs by conditional knockout of *Olig2* (a key regulator of oligodendrocyte development) in the developing cortex caused ventrally derived oligodendrocytes to migrate dorsally, but this was insufficient for achieving complete axonal myelination (Yue et al. 2006).

### 3.2 Collaborative Development of OPCs With Other Cells

During cerebral development, OPCs and GABAergic interneurons, the two distinct neural cell types, can share common developmental contexts and be in close communication with each other (Bucher et al. 2021; Liu et al. 2024; Mazuir et al. 2021). Both cell types originate from the same precursor populations within the ventricular zone, for exmple, MGE, and are regulated by similar transcriptional networks. They migrate tangentially from the ventricular zone to their final destinations, where they form synaptic connections and engage in bidirectional communication to facilitate mutual maturation (Fang et al. 2022; Orduz et al. 2019). For instance, MGE‐derived interneurons secrete fractalkine to stimulate cortical oligodendrogenesis (Voronova et al. 2017).

Developing vasculature is used as a scaffold by OPCs for migration (Duncan and Emery 2023; Tsai et al. 2016). Disruption to vascular development can inhibit ventral‐to‐dorsal OPC migration, leading to abnormal OPC accumulation in the ventral regions of the spinal cord and brain (Tsai et al. 2016). Endothelial cells secrete chemokine CXCL12 to interact with its receptors on OPCs, thereby regulating OPC migration. In the developing forebrain, MGE‐derived OPCs and interneurons are both attracted by CXCL12 released from the vasculature (Lepiemme et al. 2022). However, OPCs are believed to play a critical role in preventing direct contact between interneurons and blood vessels through a process called unidirectional contact repulsion. This mechanism steers interneuron migration away from blood vessels and directs them to follow cortical CXCL12 gradients to reach their eventual cortical layers (Lepiemme et al. 2022). As the vasculature matures, astrocytic endfeet envelop blood vessels, halting OPC perivascular migration, releasing OPCs from the vasculature. The resulting insulation of OPCs from endothelial niches that inhibit their maturation allows for their subsequent differentiation (Duncan and Emery 2023; Su et al. 2023; Wang et al. 2024).

## 4 Adult Oligodendrogenesis

**FIGURE 4:**
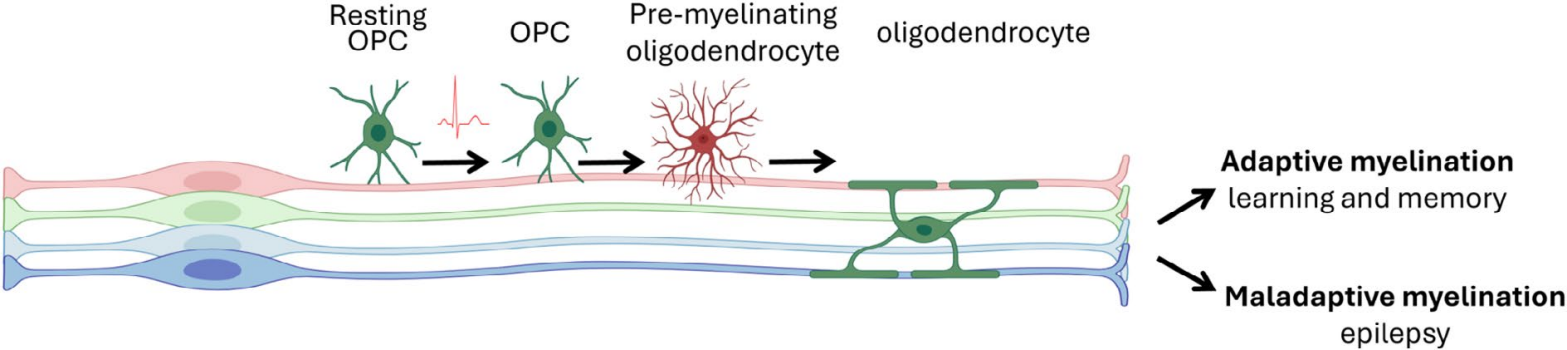
Oligodendrogenesis in the adult brain. Neuronal electrical signals stimulate the differentiation of OPCs, resulting in oligodendrogenesis and localized myelin formation. Adaptive myelination, driven by experience‐induced neuronal activity, contributes to neuroplasticity, learning and memory. In contrast, maladaptive myelination, triggered by abnormal neuronal activity, may perpetuate dysfunctional neural circuits, underlying disorders such as epilepsy.

Not all axons in the brain are myelinated (Saliani et al. 2017). For example, only 40% of neuronal fibers are myelinated in the pyramidal tract of the rat brain (Harding and Towe 1985). Given the crucial importance of myelin to signal transmission speed and neural circuit properties, newly myelinated axons are likely to be the dominant functional elements of novel brain structural connectivity. In adaptive myelination, new oligodendrocytes are generated to myelinate previously unmyelinated axons or remodel myelin sheaths, thus fine‐tuning neural circuits in response to neuronal activity, consequently contributing to neural plasticity, learning, and memory. This activity‐dependent myelination is supported by data from magnetic resonance imaging studies showing that white matter microstructure changes in task‐relevant tracts when people learn complex motor tasks such as juggling or piano playing (Bengtsson et al. 2005; Scholz et al. 2009). Growing evidence suggests that adaptive oligodendrogenesis and neo‐myelination are required for motor skill learning, working memory training, consolidation of long‐term fear and spatial memory, and opioid reward (Bacmeister et al. 2020; McKenzie et al. 2014; Pan et al. 2020; Shimizu et al. 2023; Steadman et al. 2020; Xiao et al. 2016; Yalcin et al. 2024). These studies, taken together, indicate that adult oligodendrogenesis is integral to experience‐dependent neuroplasticity and brain function (Figure 4).

### 4.1 Impact of Electrical Activity on Adult Oligodendrogenesis

OPCs can form glutamatergic and GABAergic synapses with axons, receiving synaptic inputs from neurons (Bergles et al. 2000; Gallo et al. 1996; Káradóttir et al. 2008; Kukley et al. 2007; Lin and Bergles 2004; Patneau et al. 1994). OPCs seem able to sense axonal electrical activity and respond by differentiating into myelinating oligodendrocytes, preferentially targeting active axons (Nishiyama et al. 2021). In an early study, reducing neuronal activity, such as through tetrodotoxin administration or optic nerve transection, decreased OPC proliferation (Barres and Raff 1993). Conversely, electrical stimulation of corticospinal axons in the dorsal corticospinal tract enhanced OPC proliferation and differentiation (Li et al. 2010). Moreover, optogenetic stimulation of the premotor cortex could promote oligodendrogenesis and myelination in the deep layers of the premotor cortex and subcortical white matter, resulting in improved limb motor function (Gibson et al. 2014). Chemogenetic activation of somatosensory axons also proved able to increase both OPC proliferation and differentiation in the underlying white matter (Mitew et al. 2018). In addition, genetic ablation of AMPA receptor (AMPAR) subunits GluA2, GluA3, and GluA4 in OPCs—to prevent AMPAR‐mediated synaptic inputs to OPCs—led to reduced oligodendrocyte survival in the subcortical white matter and a decrease in myelin formation, highlighting the influence of neuron‐OPC synaptic interaction on oligodendrogenesis (Kougioumtzidou et al. 2017).

### 4.2 Impact of Intercellular Molecular Mechanisms on Adult Oligodendrogenesis

Initial electrical activity may not be able to directly induce OPC differentiation and adaptive myelination but instead serves to modulate other cells to initiate molecular mechanisms that promote oligodendrogenesis (Pease‐Raissi and Chan 2021). Several intercellular molecular pathways have been implicated in adaptive myelination (Knowles, Batra, et al. 2022; Pease‐Raissi and Chan 2021; Simons et al. 2024). For example, in a mouse model of chemotherapy‐related cognitive impairment, methotrexate (MTX) exposure blocked myelination of cortical projection neurons, causing cognitive deficits. This MTX effect was linked to reduced cortical expression of BDNF. Remarkably, treatment with a TrkB agonist rescued both the myelination defect and cognitive impairment, with the rescue depending on the expression of BDNF receptor TrkB in OPCs, demonstrating the important role of BDNF–TrkB signaling in activity‐dependent oligodendrogenesis (Geraghty et al. 2019).

In another study using forced swimming stress in mice, stress triggered the secretion of dynorphin by unmyelinated axons in the striatum, which in turn stimulated OPC differentiation and myelination of nearby axons, and blocking dynorphin receptor KOR pharmacologically or through gene deletion suppressed this experience‐dependent oligodendrogenesis (Osso et al. 2021).

Interestingly, a link between decreased numbers of myelin sheaths in the prefrontal cortex and reduced vascular endothelin expression was found in mice following social isolation (Swire et al. 2019), and enhancing endothelin signaling was able to rescue this myelination defect (Swire et al. 2019), implying that vasculature can respond to experience‐related neuronal activity to influence myelination, possibly through increased metabolic demand triggered by neuronal activity (Knowles, Batra, et al. 2022; Swire et al. 2019).

### 4.3 Non‐Canonical Adult Oligodendrogenesis

A recent study demonstrated adult oligodendrogenesis in the median eminence (ME), particularly its role in maintaining energy balance in a fasting‐refeeding paradigm (Kohnke et al. 2021). The ME, which is a circumventricular organ lacking the typical blood–brain barrier, located at the ventral base of the hypothalamus, is essential to homeostatic functions including energy regulation. In the ME, the OPC proliferation rate is appreciably higher than in adjacent hypothalamic regions (Robins et al. 2013; Zilkha‐Falb et al. 2020). Although the overall number of oligodendrocytes and myelin remain stable in the adult ME, oligodendrocytes there have an unusually high turnover rate and are continuously and rapidly generated (Buller et al. 2023). It was shown that refeeding after overnight fasting triggered OPC proliferation and differentiation specifically in the ME, profoundly impacting on the formation and remodeling of perineuronal nets (Kohnke et al. 2021). In addition, adult oligodendrogenesis in the ME was quickly stimulated by hypoglycaemia (Buller et al. 2024). Intriguingly, newly generated oligodendrocytes in the ME were found to engage in regulating glucose homeostasis and maintaining the integrity of the blood‐hypothalamus barrier not through myelin production but via an ADAMTS4‐mediated mechanism (Buller et al. 2024). Given that the brain contains seven circumventricular organs (referred to as the “seven windows of the brain”) (Gross et al. 1987), further research into oligodendrogenesis in these unique brain regions could offer deeper insights into its roles in systemic homeostasis and neurovascular interaction.

## 5 Oligodendrogenesis in Aging and Diseased Brain

Oligodendrogenesis and myelination persist throughout life, though at a declining rate with increasing age (Kasuga et al. 2019; Psachoulia et al. 2009; Young et al. 2013). In aging rodents and non‐human primates, studies have revealed accumulation of abnormal myelin microstructures and significant loss of white matter volume (Groh and Simons 2025; Peters 2009; Peters and Kemper 2012). These findings suggest that aged oligodendrocytes may undergo cell death or lose their ability to produce and maintain functional myelin sheaths, while newly generated oligodendrocytes in older individuals often fail to form structurally normal myelin, exacerbating age‐related white matter deterioration. Apart from aging, disruption to oligodendrogenesis and damage to myelin integrity are important contributors to various neurological conditions, including AD, epilepsy, and movement disorders (Groh and Simons 2025; Knowles, Batra, et al. 2022; Song et al. 2024).

### 5.1 Oligodendrogenesis in Aging

The impact of aging on OPCs and oligodendrogenesis has been a topic of debate. Some studies report no changes in OPC numbers and proliferation in the aging brain, while others indicate a decline in both aspects. Nonetheless, it is widely accepted that oligodendrogenesis decreases with age in rodents and mammals (Dimovasili et al. 2023; Kasuga et al. 2019; Segel et al. 2019; Spitzer et al. 2019; Wang et al. 2020; Yeung et al. 2014; Young et al. 2013). This decline has been confirmed through in vivo imaging in aged mice showing a significant reduction in pre‐myelinating oligodendrocytes and myelination. (Hill et al. 2018). Reduced OPC proliferation has also been observed in the aged brain and attributed to decreased expression of key factors such as PDGFRA (Seeker et al. 2023; Windener et al. 2024). Diminished OPC differentiation with aging likely arises from a combination of factors including dysregulated transcriptional networks, epigenetic alterations, the onset of cellular senescence, altered neuronal activity, energy inefficiency in the brain, and oxidative stress (Aber et al. 2022; Luan et al. 2021; Ma et al. 2022; Neumann et al. 2019; Sams 2021; Shen et al. 2008; Spitzer et al. 2019; Zhao et al. 2022). In addition, increased stiffness of brain ECM is also thought to be responsible for the deterioration of OPC function in aging (Segel et al. 2019). Curiously, studies in aged mice and gray mouse lemurs revealed an increase in oligodendrogenesis and myelination in the subventricular zone (SVZ), although this region has little influence on the overall population of adult‐born oligodendrocytes (Butruille et al. 2023; Capilla‐Gonzalez et al. 2015). Recent single‐cell RNA sequencing data provide a new perspective on aging‐related changes in OPCs. Aging OPCs adopt a transcriptomic profile characterized by an inflammatory and differentiation‐inhibiting state, with increased HIF‐1α and WNT signaling as well as upregulated complement expression (Heo et al. 2024). Similarly, a significant proportion of aged oligodendrocytes in the white matter transition into a disease‐associated state. These aged cells feature upregulation of pathways relating to cellular stress or immune signaling and downregulation of those involved in cholesterol biosynthesis, hinting at a shift in function from supporting and maintaining myelin to prioritizing stress response pathways and survival (Groh and Simons 2025; Kaya et al. 2022).

### 5.2 Oligodendrogenesis in AD

The breakdown of myelin sheaths has been considered as an early pathological phenomenon of AD (Bartzokis 2004; Dean III et al. 2017), and significant white matter alterations often occur in the AD brain (Benitez et al. 2014; Phillips et al. 2016; Roseborough et al. 2017). Recent data suggest that myelin defects in aging may stimulate amyloid‐β plaque formation, hence being proposed as an upstream AD risk factor (Depp et al. 2023). The breakdown of myelin would follow a pattern mirroring the process of myelination during development, but oligodendrogenesis in AD is likely impaired since OPCs appear particularly vulnerable to a deteriorating microenvironment, which can weaken OPCs' ability to differentiate into functional oligodendrocytes (Bartzokis 2004; Zou et al. 2023).

It is important to note that unlike AD progression in humans, AD mouse models generated by overexpressing AD‐related mutant gene(s), including APP, MAPT, and PSEN1, start to express the transgenes from developmental stages and develop pathological changes at an early age, meaning a younger and more responsive state for OPCs, likely impacting neuronal development per se in these mice. Increased OPC proliferation and differentiation were found in the APP/PS1 mouse model of AD during a specific time window, between 6 and 8 months of age (Behrendt et al. 2013). Another study using the same model discovered a significant rise in newly generated oligodendrocytes and myelination at 10 months of age (Chen et al. 2021). The number of newly formed oligodendrocytes was also elevated in the hippocampus, entorhinal cortex, and fimbria of 6‐month‐old J20 mice, another AD mouse model (Ferreira et al. 2020). In the 5XFAD AD mouse model, OPC proliferation and the numbers of pre‐myelinating and newly matured oligodendrocytes were increased as well (Kedia et al. 2024) Despite such increases in OPC activity—likely driven by OPCs' response to local demyelination—the overall myelin levels in the cortex and hippocampus of APP/PS1 mice, as well as in postmortem tissues of AD patients, have been shown to be significantly decreased, and myelin structural changes have been observed in APP/PS1, J20, and 5XFAD mice (Chen et al. 2021) (Behrendt et al. 2013) (Ferreira et al. 2020) (Kedia et al. 2024). Remarkably, boosting myelination through genetic or pharmacological interventions was able to restore myelin levels and improve spatial learning deficits in APP/PS1 mice (Chen et al. 2021). Additionally, single‐cell RNA sequencing of prefrontal cortex tissues from AD patients has revealed unexpected myelin‐related gene expression across multiple cell types, possibly as a response to myelin loss (Mathys et al. 2019). These findings underscore the important role of oligodendrogenesis in AD pathology and highlight its potential as a promising target for therapeutic strategies aimed at mitigating AD progression.

### 5.3 Maladaptive Myelination

The term “maladaptive myelination” refers to a pathological state where oligodendrogenesis along with myelin alters, driven by abnormal neuronal activity, exacerbating neuronal dysfunction rather than compensating for it (Knowles, Batra, et al. 2022; Knowles, Xu, et al. 2022; “Maladaptive myelination promotes seizure progression in generalized epilepsy,” 2022). Unlike impaired myelination, maladaptive myelination involves activity‐dependent myelin plasticity and results in reinforcing the dysfunctional neural networks that induced it (Figure 4). In rodents with absence seizures, an increase in OPC proliferation, mature oligodendrocyte numbers and myelin sheath thickness in the corpus callosum was observed after epilepsy onset, specifically in regions involved in seizure propagation (Knowles, Xu, et al. 2022). Blocking activity‐dependent myelination prevented seizure progression, suggesting that maladaptive myelination could create a feedback loop, sustaining seizures (Knowles, Xu, et al. 2022). Similarly, maladaptive myelination may underlie movement disorders. In addition, neuroimaging studies have demonstrated altered white matter structure in conditions like idiopathic dystonia (Bonilha et al. 2007; Kim et al. 2015) and Tourette syndrome (Atkinson‐Clement et al. 2020), hinting at impaired myelin plasticity although little is known about the precise mechanisms.

## 6 Concluding Remarks

The invention of myelin was a momentous milestone in vertebrate evolution, bringing about highly efficient neural computation within the confined space of the CNS. Over the past couple of decades, advancements in genetic analysis, next‐generation sequencing, and imaging technologies have significantly deepened our understanding of oligodendrogenesis in respect to its evolutionary origin, role in development, contribution to neural plasticity, and involvement in pathological conditions. Notably, the concepts of adaptive and maladaptive myelination have expanded our knowledge of brain function and dysfunction.

Despite these advances, many interesting questions remain unanswered—for instance: How did *Chondrichthyes* evolve regulatory networks for oligodendrogenesis and myelination? What myelin proteins (apart from P0) transition from the CNS to PNS during amphibian metamorphosis, and how? What are the functional roles of PDGFRA‐negative OPCs in the CNS of mammals? What are the molecular driving forces behind adult oligodendrogenesis and adaptive myelination? Is maladaptive myelination a common phenomenon in pathological conditions? Can interventions targeting adult oligodendrogenesis, adaptive myelination, or maladaptive myelination provide new therapeutic avenues for neurological diseases? Addressing these questions will be crucial to gaining fresh insights into evolution, CNS function and disorders.

## Author Contributions

H.H., T.G., J.Z., and H.L. drafted the manuscript. H.L. and J.Z. finalized the article.

## Conflicts of Interest

The authors declare no conflicts of interest.

## Data Availability

Data sharing is not applicable to this review as no datasets were generated or analysed during the current study.
